# Piezoelectric Energy Harvesting from Suspension Structures with Piezoelectric Layers

**DOI:** 10.3390/s20133755

**Published:** 2020-07-04

**Authors:** Min Wang, Yiming Xia, Huayan Pu, Yi Sun, Jiheng Ding, Jun Luo, Shaorong Xie, Yan Peng, Quan Zhang, Zhongjie Li

**Affiliations:** 1 School of Mechatronic Engineering and Automation, Shanghai University, Shanghai 200444, China; xmwangmin@shu.edu.cn (M.W.); xym429@shu.edu.cn (Y.X.); phygood_2001@shu.edu.cn (H.P.); yisun@shu.edu.cn (Y.S.); ding_jiheng@shu.edu.cn (J.D.); luojun@shu.edu.cn (J.L.); srxie@shu.edu.cn (S.X.); pengyan@shu.edu.cn (Y.P.); lincolnquan@shu.edu.cn (Q.Z.); 2 Shanghai Institute of Intelligent Science and Technology, Tongji University, Shanghai 200092, China; 3 Department of Mechanical and Industrial Engineering, University of Toronto, Toronto, ON M5S 3G8, Canada

**Keywords:** suspension structures, piezoelectric ceramics, energy harvesting, low-frequency vibration system, bending mode

## Abstract

In this paper, we propose a generator for piezoelectric energy harvesting from suspension structures. This device consists of a leaf spring and eight pairs of piezoelectric layers attached to inner and outer surfaces. We present a special type of leaf spring, which can magnify the force from the workload to allow the piezoelectric layers to achieve larger deformation. The generator is to solve the problem of vibration energy reutilization in a low-frequency vibration system. To verify the efficiency of the proposed configuration, a series of experiments are operated. The results indicate that the resonance frequency (25.2 Hz) obtained from the sweep experiment is close to the simulation result (26.1 Hz). Impedance-matching experiments show that the sum of the output power attains 1.7 mW, and the maximum single layer reaches 0.6 mW with an impedance matching of 610 KΩ, and the instantaneous peak-peak power density is 3.82 mW/cm^3^. The capacitor-charging performance of the generator is also excellent under the series condition. For a 4.7 μF capacitor, the voltage is charged to 25 V in 30 s and limited at 32 V in 80 s. These results demonstrate the exploitable potential of piezoelectric energy harvesting from suspension structures.

## 1. Introduction

Energy harvesting has remained a major focus of researchers in different fields since it was proposed approximately twenty years ago [1]. One of the main motivations of energy harvesting is to reduce the amount of chemical waste produced by the extensive use of fuel cells, which not only protects the ecological environment but also offers potential monetary gains [2]. Harvesting has achieved considerable success in sustainable and wholescale energy deployment from solar [3], tidal [4] and hydroelectric energy sources [5], however, these three methods are not applicable for small-scale energy conversion. At a certain point, it becomes more convenient to acquire vibration-based energy from suspension structures, which are independent of natural conditions. Vibration-based energy harvesting from suspension structures usually employs four mechanisms [6]: the piezoelectric effect [7,8,9,10,11,12,13,14,15,16,17], the electromagnetic effect [18,19,20,21,22], the magnetostrictive effect [23,24] and the electrostatic effect [25,26,27,28]. Compared to the other three mechanisms, the greatest advantage of piezoelectric devices is their large power densities [2], which are on par with lithium-ion batteries.

Both the compression and bending modes [29] of piezoelectric ceramics are commonly utilized for energy harvesting from vibration-based suspension structures, which can also be called stack actuators and bimorphs, respectively. Since the former has a higher coupling factor, which means higher energy conversion, it has attracted a vast amount of studies. Li et al. designed a hybrid generator applied to low-frequency ambient vibrations for energy harvesting, conducted experiments under strong compressive operation modes, and achieved a maximum power of 19.6 mW [30]. Zhang et al. analyzed the nonlinear theory of a piezoelectric vibrational energy harvester and established the nonlinear spring-back model [31]. Qian et al. presented a distributed-parameter model of an axial vibration-based multilayer piezoelectric stack transducer with a connecting rod, and validated its accuracy and reliability by experiments [32]. Feenstra et al. proposed a novel backpack by importing a mechanically amplified stack to generate electrical energy from the pressure difference between the pack and the wearer, and the experiments showed that this system could obtain a mean power of 0.4 mW [33]. Su et al. established the dynamic model of a horizontal rotating piezoelectric energy harvester and the experimental results show that the model has good stability [34]. Gljušćić et al. assessed the power requirements of wearable sensors for medical applications and studied excitation patterns aiming at increasing specific power output [35]. Hendrowati et al. established the mathematical model of a multilayer piezoelectric vibration-based energy harvesting mechanism and produced an output voltage of 2.75 V when mounting with a spring, and the power output was 7.17 times greater than a mechanism without a spring [36]. Zhao et al. investigated the numerical solutions of a multilayer piezoelectric stack configuration under uniaxial dynamic pressure loading and verified the validity of the model through experiments under harmonic excitations at different pressure levels [37]. Wang et al. studied theoretical models of piezoelectric energy harvesting using stack-type and patch-type piezoelectric transducers in railway systems and proved that piezoelectric transducers could not only harvest the available energy from the vibration of the track but also serve as sensors to continuously monitor the train [38].

However, in compression mode, only a very high workload can produce a considerable energy harvesting effect, and the device size must also be sufficiently large, which limits the energy harvesting of small structures or very low workloads. In these cases, bending mode conversion has advantages such as a very large ratio of strain to stress, which means that a small workload can produce a great strain. This is of great significance for vibration energy harvesting from suspension microstructures with small workloads. In addition, bimorphs are cheaper to manufacture but offer higher energy density. Panda et al. designed various electronic circuitries with different combinations of electronic components and found that piezoelectric bimorphs obtained an output voltage of 450 mV and multilayer stacks of 125 mV [39]. Based on the theory of Kirchhoff plate and the modal analysis of physical and modal coordinates, Koszewnik et al. established the distributed parameter electroelastic model of aluminum plate bonded by the harvester with two kinds of piezoelectric actuators and the results of the electroelastic analysis model are verified experimentally [40]. Zhou et al. presented a novel nonlinear piezoelectric energy harvesting system, composed of a linear spring-connected linear piezoelectric energy harvester and analyzed the nonlinear dynamic response [41]. Pozzi et al. presented a bimorph structure in pure bending via a compliant rotational institution with an acquired power of 3.4 mW under pure bending at a frequency of 56.7 Hz and an acceleration of 5 g; the acquired power was 1.3 mW without the compliant rotational institution [42]. Wang et al. proposed a new system by integrating multiple piezoelectric bimorphs of a series of aspect ratios to achieve broadband piezoelectric harvesting and proved that the operating frequency band could be tailored by the connection patterns [43]. El-Sabbagh et al. changed the topology of a bimorph and harvested more power by decreasing the thickness of bimorphs at anti-nodal elements by allowing additional straining [44]. He et al. presented vibration energy harvesters with a rolling steel ball inside a guiding channel as the proof mass and derived the mathematical model of the system [45]. Abramovich et al. established an analytical model of three bimorphs with three end masses and obtained power levels up to 20 and 5 mW at the first and second natural frequencies, respectively [46]. Wang et al. developed evaluation methods with an output capacity density indicator and obtained good agreement between simulations and analytical result [47]. Pozzi et al. designed a compact and low-profile wearable energy harvesting device and recorded a power output of 50 mW for every walking step and 70 mW for every running step [48]. Zhao et al. performed electroelastic modelling and experiments on a piezoelectric energy harvester based on broadband random vibrations, and the results achieved a high level of agreement [49]. Pozzi et al. proposed a piezoelectric energy harvester based on the plucking technique to be worn on the knee joint and obtained a power output of 2.06 ± 0.3 mW [50]. Bonello et al. utilized a piezoelectric vibration energy harvesting beam to suppress a particular vibration mode of a tuned mass damper prototype and achieved the ideal degree of vibration attenuation [51]. Zhang et al. analysed a frequency-adjustable energy harvester that was successfully operated at multiple frequencies, which could be adjusted by the spring stiffness [52]. Hosseini et al. deduced a precise and concise formula to calculate the frequency of bimorphs and concluded that a triangular cantilever has the highest power density [53]. Cottone et al. presented a theoretical model of a nonlinear vibration energy harvester and compared the bandwidth and output power between monostable and bistable regimes under an optimal acceleration level [54]. Priya et al. demonstrated a piezoelectric windmill utilizing 12 bimorphs and obtained a power output of 10.2 mW at a frequency of 6 Hz [55]. Pozzi et al. developed an analytical model of a piezoelectric bimorph based on the Euler–Bernoulli beam and defined the dimensionless variables and parameters [56]. Benasciutti et al. studied piezoelectric resonant bimorph beams for vibration energy harvesting and optimized the structures to improve the performance of the prototype [57]. Chandrasekharan et al. investigated the possibility of integrating lightweight honeycomb structures with piezoelectric bimorphs to obtain a higher specific power [58]. Aktakka et al. reported an energy harvester that generated energy from the wing motion of insects during their flights and utilized piezoelectric bimorphs operating in the 31-mode. The harvester produced a power output of 18.5–22.5 µW in the simulations [59].

In this paper, we propose a generator for piezoelectric energy harvesting from suspension structures. The main contributions are as follows: first, the leaf spring is put to use on the suspension structures for the first time, with which the resonant frequency of the generator can be designed to be as low as that of an automobile engine; second, with the multi-plane of different dimensions of the leaf spring, vibration energy in all directions is harvested to the maximum. At the same time, the magnitude of the energy at different locations can also be compared; third, the sum of the 16 piezoelectric layers’ instantaneous peak-peak power reaches 1.7 mW, and the instantaneous peak-peak power density is 3.82 mW/cm^3^; finally, an excellent capability was demonstrated to charge capacitances at the micro level.

## 2. Design and Simulation of the Generator

**Figure 1:**
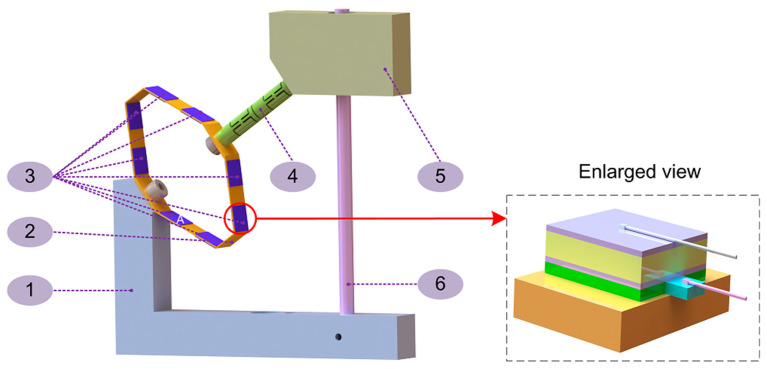
The overall schematic diagram of the generator and the enlarged view of the piezoelectric layer. Key components are listed as follows: 1. Base. 2. Leaf spring. 3. Piezoelectric layers. 4. Flexible hinge. 5. Workload. 6. Linear bearing guide rod.

The overall schematic diagram of the generator, shown in Figure 1, is composed of four components: the piezoelectric layers, the bearing system, the base and the workload. The leaf spring is fixed to the base with a bolt and a nut. Eight pairs of piezoelectric layers are attached to the spring with a structural adhesive with an enlarged view to show details. The guide rod of the linear bearing is inserted into the base through a hole in the base that is slightly larger than the guide rod, and a bolt is screwed in the vertical direction to completely restrain the guide rod. The linear bearing is attached to the mass with a structural adhesive. The guide rod passes through the linear bearing to achieve the vertical linear motion of the mass. Both ends of the flexible hinge are connected by threads to the mass and the leaf spring. For clarity and convenience, we marked the position on the spring next to the bolt as “A” and numbered the outside piezoelectric 1 to 8 from position “A” in a counterclockwise direction. The piezoelectric layers number 1 to 8 correspond to the inner piezoelectric layers numbered 9 to 16, respectively. The piezoelectric ceramic (yellow part) is sandwiched between two thin silver coatings (violet parts), and the silver layers of each piezoelectric layer are marked with a positive or a negative electrode. The negative electrodes of the piezoelectric layers number 1 to 8 are bonded to the spring with structural adhesive (green part). To output the voltage generated when the piezoelectric plate is deformed, we derive two wires from the silver layers of each piezoelectric layer. The silver layer bonded to the spring is connected to the wire by drawing out a layer of 0.5-micron-thick copper foil, and the wire is welded to the copper foil, while the other wire is welded directly to the silver.

**Figure 2:**
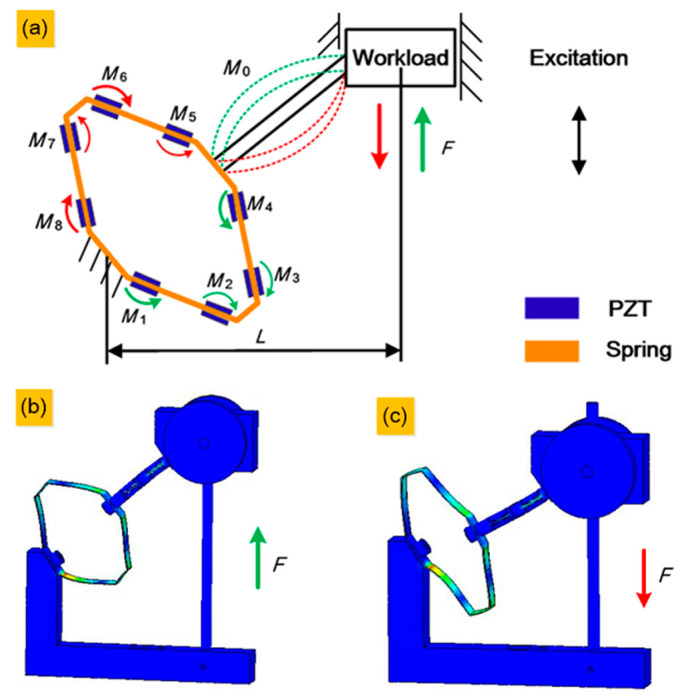
Force transmission in the generator: (**a**) Theoretical analysis. (**b**) Static simulation with vertical upward force. (**c**) Static simulation with vertical downward force.

When the generator is excited vertically, the workload reciprocates with the linear bearing along the bearing guide rod, producing a vertical force on the system. Considering the inevitable assembly accuracy problems, small torques may be produced in the direction perpendicular to the cross-section of the spring. Therefore, a flexible hinge is introduced into the system to avoid the distortion of the spring caused by this small torque. In accordance with a study by Xi’an Jiaotong University in 2005, four plates with piezoelectric layers of the leaf spring have the same mode shape, whose two non-free edges remain in a straight line [60]. This means that the force applied to each plate of the spring can be expressed, as shown in Figure 2a. The spring serves to magnify the force from the workload to allow the piezoelectric layers to achieve a larger deformation. Denoting the force produced by the up–down movement of the workload as *F*, the force equilibrium equation of the system can be expressed as

(1) $$F*L=M_{0}+M_{1}+M_{2}+M_{3}+M_{4}+M_{5}+M_{6}+M_{7}+M_{8}$$

where *F*, *L*, $M_{0}$, and $M_{1-8}$ are the force on the system from the workload, horizontal static distance from the centre of the workload to the fulcrum, torque of the flexure hinge and torque of each position on the spring, respectively, and the subscript of the torque symbol corresponds to the labelled number of the position. F∗L represents the total torque input to the system. Particularly, each torque is in the opposite direction when the workload goes down compared to when it goes up. To avoid mixing, we omit the torque of the upper half of the spring in the upward movement (the green parts) and the lower half in the downward movement (the red parts). Figure 2b shows the static simulation result of a vertical upward force, corresponding to the green parts in Figure 2a. Figure 2c shows the static simulation result of a vertical downward force, corresponding to the red parts in Figure 2a. The static stress simulation results show that the strain varies from position to position, which is reflected by the thickness of the arrow in Figure 2a. According to the static simulation results in Figure 2b,c, we infer that position number 1 will harvest the highest output voltage cause red means the maximum stress, and positions number 2 and 6 will harvest the lowest output voltage. Because blue means the minimum stress, the rest of the positions will be in the medium range, with green, in the Figure 2b,c.

**Figure 3:**
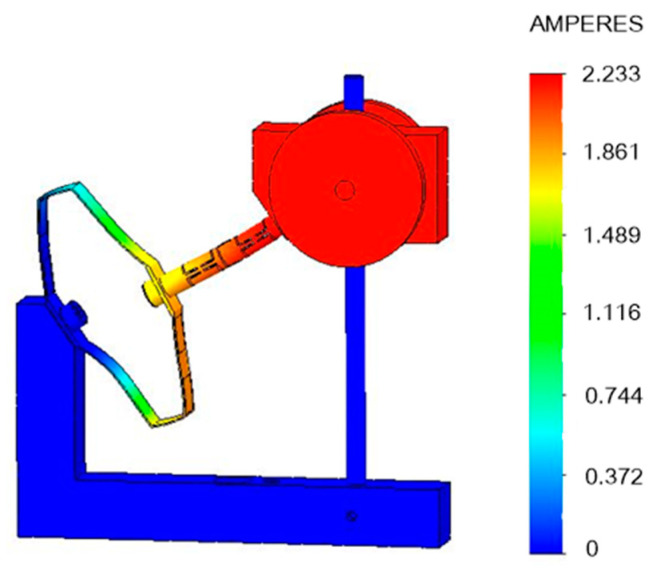
Simulation result of the first mode frequency.

Finally, the resonance frequency is obtained by the frequency simulation, in which the first mode measures 26.066 Hz, as shown in Figure 3. The strain at each point of the spring is represented by different colors. On the right side of Figure 3, the values of the strain are listed, corresponding to each color. All the simulations above were completed by SOLIDWORKS 2016, Dassault Systems.

## 3. Experiments and Discussion

In this section, we designed a series of experiments to assess the performance of the generator. Impedance-matching experiments show that the sum of the output power attains 1.7 mW, the maximum single layer reaches 0.6 mW, with an impedance matching of 610 kΩ, and the instantaneous peak-peak power density is 3.82 mW/cm^3^. The capacitor-charging performance of the generator is also excellent under the series condition. For a 4.7 μF capacitor, the voltage is charged to 25 V in 30 s and limited at 32 V in 80 s. These results demonstrate the exploitable potential of piezoelectric energy harvesting from suspension structures.

### 3.1. Prototype Fabrication and Experimental Setup

**Figure 4:**
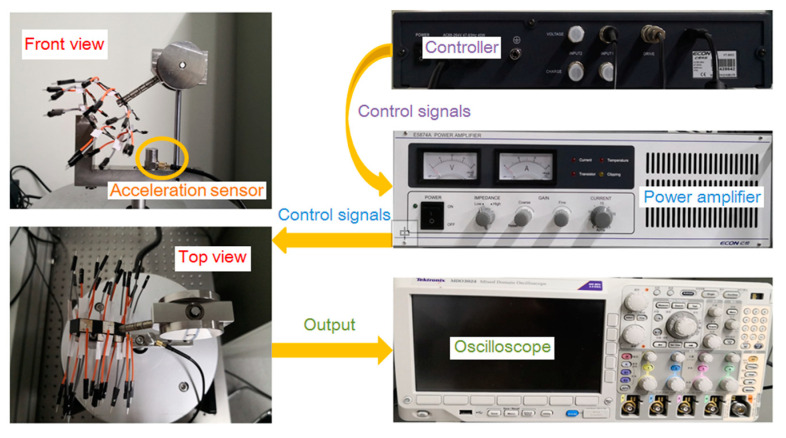
The fabricated prototype of the energy harvesting system.

The prototype shown in Figure 4 was processed according to the design shown in Figure 1. Figure 4 shows the front and top view of the prototype. We designed a series of experiments to evaluate the performance of the prototype. On the side away from the spring, each piezoelectric layer is welded with a wire as one of the electrodes, and there is a small, thin piece of copper foil between each piezoelectric layer and spring as the other electrode. The thickness of the copper foil layers is 0.05 mm. The dimensions of the piezoelectric layers are 9 × 9 × 0.35 mm, made by piezoelectric-5H. Two silver layers covering the upper and lower surfaces of each piezoelectric layer act as electrodes.

The experiments are performed on a shaker (E-JZK-50, ECON Technologies Co., Ltd., Hangzhou, China) powered by an amplifier (E5874A, ECON Technologies Co., Ltd.), as shown in Figure 4. This provides simple harmonic motion to the prototype and is controlled by a vibration controller (VT-9002, ECON Technologies Co., Ltd., Hangzhou, China). Experimental parameters, such as acceleration and sweep time, are fed into the amplifier through vibration control software in a computer, and an acceleration sensor (EA-YD-181, ECON Technologies Co., Ltd., Hangzhou, China) fixed to the prototype feeds the acceleration signal back to the controller. An oscilloscope (MDO3024, Tektronix, Oregon, OR, USA) has four channels that ensure that the frequency signal of the four piezoelectric layers can be displayed simultaneously. Other material properties and prototype parameters are listed in Table 1, and the resonance frequency of the prototype is 26.07 Hz according to the simulation results.

### 3.2. Experimental Results and Discussion

#### 3.2.1. Voltage Responses from Frequency Sweeps and Voltages at Resonance

**Figure 5:**
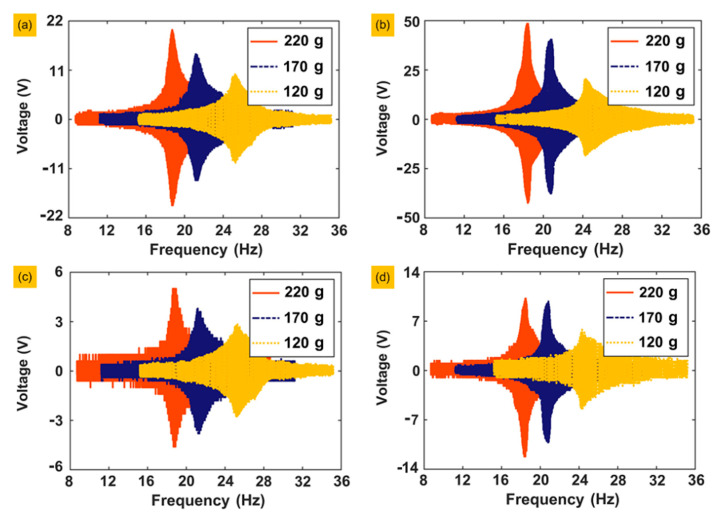
Voltage responses of frequency sweeping: (**a**) Voltage responses of piezoelectric layer number 1, with acceleration of 0.1 g under different masses. (**b**) Voltage responses of piezoelectric layer number 1, with acceleration of 0.2 g under different masses. (**c**) Voltage responses of piezoelectric layer number 3, with acceleration of 0.1 g under different masses. (**d**) Voltage responses of piezoelectric layer number 3, with acceleration of 0.2 g under different masses.

The first group of experiments is run to determine the actual resonance frequency of the prototype through frequency sweeps. We selected two of the piezoelectric layers, 1 and 3, and set the frequency domain as (8 Hz, 36 Hz) with a frequency sweep rate of 0.1 Hz/s. For each piezoelectric layer, the weights of the three groups of masses were 120, 170 and 220 g with accelerations of 0.1 and 0.2 g, respectively (g is the gravity acceleration and g = 9.82 m/s^2^). Each experiment is conducted under open-circuit conditions. The experimental results of frequency sweeping are shown in Figure 5.

**Figure 6:**
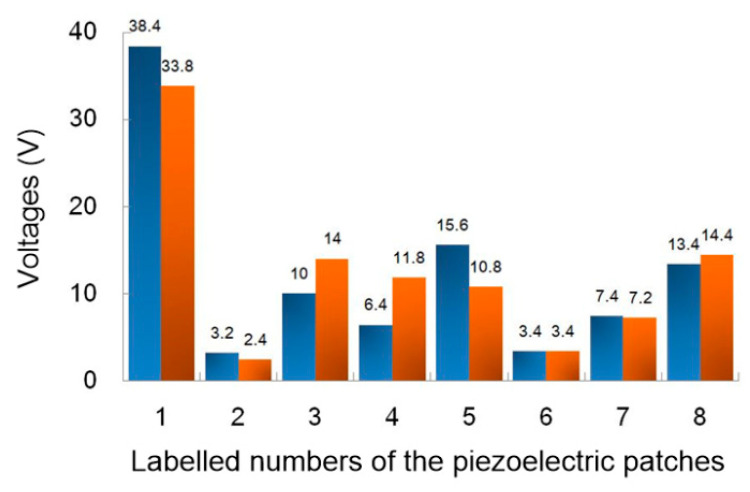
The output voltage of the eight piezoelectric patches.

According to the results of frequency sweeping, the resonance frequencies of the prototype are 19.8 and 25.2 Hz under masses of 220, 170 and 120 g. Then, under a mass of 120 g and an acceleration of 0.2 g, we measured the output voltage of all sixteen piezoelectric layers, as shown in Figure 6. Experimental results show that the output voltages on positions 2 and 6 are both the lowest, while position 1 is the highest, and positions 3, 5 and 8 are in the medium range, which is highly consistent with the simulation results of static stress.

**Figure 7:**
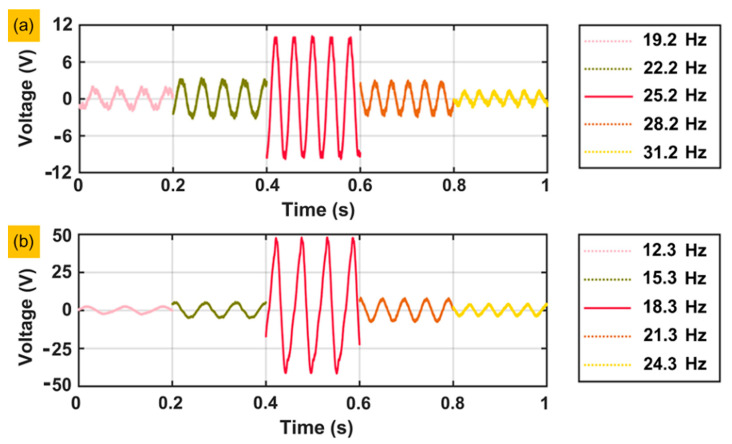
The output voltage performance of the piezoelectric layer labelled number 1 at the exact frequencies: (**a**) Under a mass of 120 g and an acceleration of 0.1 g. (**b**) Under a mass of 220 g and an acceleration of 0.1 g.

Figure 7 shows the output voltage performance of piezoelectric layer 1 under the open-circuit condition and presents the amplitude of the output voltage under different excitation frequencies. Figure 7a is under the condition with a mass of 120 g and an acceleration of 0.1 g. The highest amplitude of the output voltage appears at a resonance frequency of 25.2 Hz. Figure 7b is under the condition with a mass of 220 g and an acceleration of 0.1 g. The highest amplitude of the output voltage appears at a resonance frequency of 18.3 Hz. Both conditions show that the output voltage decreases with increasing or decreasing excitation frequency. The amplitude of the output voltage drop is positively correlated with the distance between the excitation frequency and resonance frequency.

#### 3.2.2. Impedance Matching and Output Power

**Figure 8:**
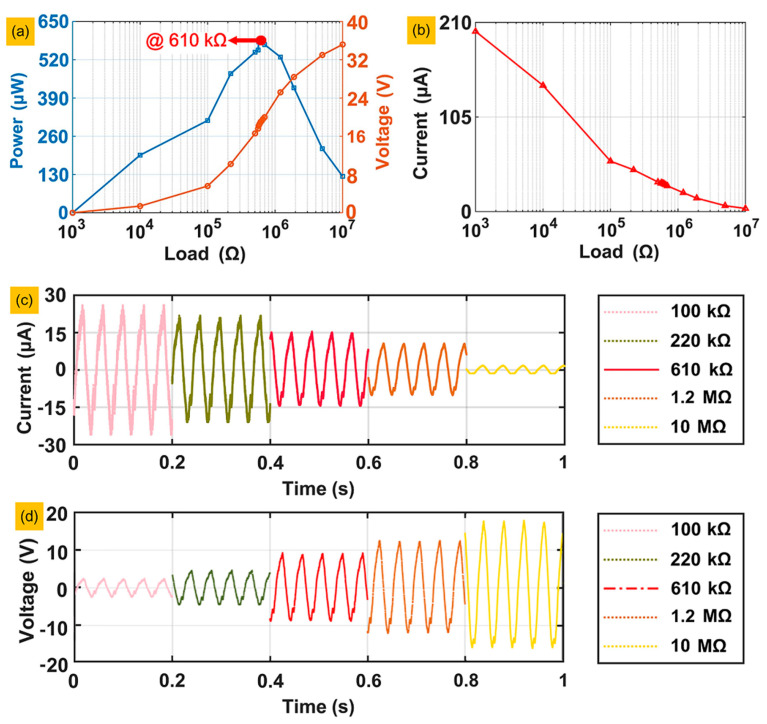
The power, output voltage and current of piezoelectric layer number 1 matching different impedances: (**a**) The power and output voltage curves. (**b**) The current curve. (**c**) Current signals under different circuit load conditions. (**d**) Voltage signals under different circuit load conditions.

This section aims to evaluate the amount of energy generated by the deformation of the piezoelectric layers, we tested the output power of piezoelectric layer number 1 in series with different resistors from 1 kΩ to 10 MΩ under a mass of 120 g and an acceleration of 0.2 g. According to Ohm’s law, $P_{P-P}=\frac{U_{P-P}{}^{2}}{R}\;$, in which *R*, $U_{P-P}$ and $P_{P-P}$ are the external resistance, peak–peak voltage and power of the external resistance, respectively, the values of each group of *R*, $U_{P-P}$ and $P_{P-P}\;$, are listed in the line chart shown in Figure 8a. When *R* of the external resistance is close to the piezoelectric layer, which is called the matched resistance, the value of $P_{P-P}$ reaches its maximum. The voltages that lead to power results were all obtained at steady states. The matched resistance is approximately 610 kΩ in Figure 8a. In the theoretical equation of the matched resistance $R_{0}=1/\left(2\pi fC\right)$, *f* and *C* are the resonant frequency of the prototype with 120 g loading and the capacitance of the piezoelectric layer, respectively. Taking the value of the capacitance as 10.2 nF and the resonant frequency of the prototype as 25.2 Hz, the theoretical value of the matched resistance is 619.18 kΩ, and the error of the experimental result is approximately 1.5%. Figure 8b shows the currents of the load circuit with the 16 groups of external resistances. As the external resistance increases, the current goes to zero and decreases evermore slowly, because, relative to the external resistance at this time, the resistance of the piezoelectric layer can be neglected. To reflect the characteristics of the current signals more clearly, we selected the current signals of five groups of resistance in the time domain and made a chart, as shown in Figure 8c, which also shows that the current decreases as the resistance increases. Figure 8d shows the voltage signals under different circuit load conditions: the voltage increases as the load resistance increases, and the output voltage reaches 19.2 V with the matched resistance of 610 kΩ, exactly half of the open circuit voltage, which is 38.4 V.

The peak power of all 16 piezoelectric layers was measured under the same experimental conditions, as shown in Table 2.

#### 3.2.3. Charging Performance

**Figure 9:**
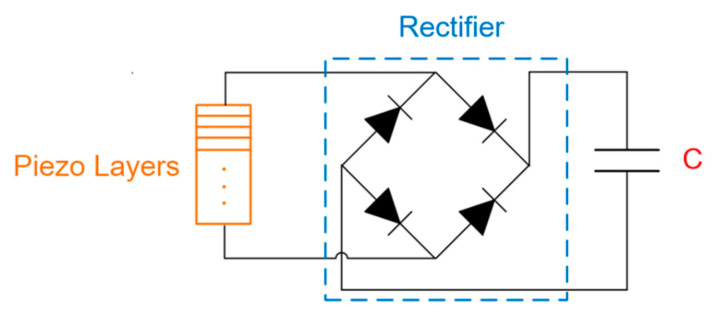
Schematic diagram of the charging circuit.

In the last set of experiments, the capacitors were charged to different capacities: 4.7, 10, 22, 33 and 47 μF, and each capacitor had a maximum voltage of 50 V. The electric energy generated by the vibration of the piezoelectric layer is rectified through a rectifying circuit and then fed into the capacitor. A schematic diagram of the charging circuit is shown in Figure 9.

**Figure 10:**
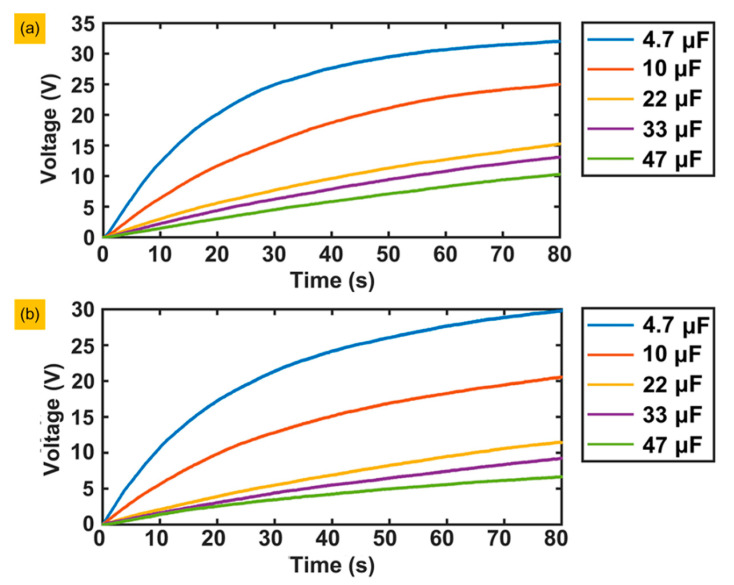
Experimental results of capacitor charging: (**a**) Series circuit. (**b**) The charging effect of only piezoelectric plate number 1.

We performed a series of 16 piezoelectric layers and a single layer charging effect comparison. In the series test, the negative pole of one piezoelectric layer was connected to the positive pole of the other until all 16 layers were connected as an integral element with positive and negative poles and then connect the positive and negative poles of the integral element to the input ends of the rectifying bridge, whose output ends are connected to the capacitor. Figure 10a shows the results of the series of experiments. The voltage can reach 25 V when the 4.7 μF capacitor charges for 30 s; after 30 s, the charging speed becomes extremely slow, and the voltage increases to 33 V after 80 s. The charging rate of the 10 μF capacitor does not decrease significantly until 50 s later. The remaining three capacitors have almost the same speed, which is much slower than the first two capacitors, and the final voltages range between 10 V and 15 V. Figure 10b shows the charging effect of piezoelectric layer number 1. The charging speed is equivalent to the 16 layers in series, but the final voltage is lower than the latter, especially for capacitors with a large capacity. All the results show that the larger the capacity is, the slower the charging speed.

## 4. Conclusions

In summary, we proposed a generator for piezoelectric energy harvesting from the suspension structures in this paper. A new model of vibration structure was proposed, which can not only obtain energy from different positions but can also combine the energy of each position according to different needs. The energy is collected through the bending patterns of the piezoelectric layers. According to the experimental results, we conclude the following:

(1) Under a force loading of 120 g and an excitation acceleration of 0.2 g at a resonant frequency of 25.2 Hz, the generator can produce a 1.7 mW peak power output with an impedance-matching of 610 kΩ, and the instantaneous peak–peak power density is 3.82 mW/cm^3^;
(2) The capacitor charging performance of the generator is also excellent under the series condition. For a 4.7 μF capacitor, the voltage is charged to 25 V in 30 s and limited at 32 V in 80 s.

The generator demonstrates excellent energy-harvesting capabilities from suspension structures. Significant voltages were obtained from different areas and voltages are correspondingly agree with the scale of stress distribution under external excitation. The average power output can reach the milliwatt level under both small loading (120 g) and acceleration (0.2 g) with impedance matching. The experimentally matched impedance is in excellent agreement with theoretical estimation. The instantaneous peak–peak power density is at the milliwatts per cubic centimeter level. The charging performance proves the feasibility of harnessing energy into stored electricity. We absolutely believe that both the structure and charging performance of the generator could be further improved by optimizing the design and the configuration of the piezoelectric layers. In future work, we will apply a mathematical model to attain the optimal position of the piezoelectric layers. Suspension systems are extensively utilized in automobiles, aircrafts, ships and industrial apparatus for vibration isolation and the vibrational energy is usually dissipated into surrounding environment. Numerous wireless sensors are embedded into these transportation media and apparatus. The expense of replacing or renewing batteries of such sensors usually costs much more than the batteries themselves. The proposed idea, which provides a solution, turning dissipated energy into electric power, can be of great significance for the further development of self-power sensing networks so as to reduce battery replacement cost.

## Figures and Tables

**Table 1:** Material properties and prototype parameters.

	Description	Value
**Piezoelectric plate (YH-52)**	Dimensions (mm)	9 × 9 × 0.35
	Density (kg/m^3^)	7450
	Piezoelectric constant d_31_ (10^−12^ C/N)	−210
	Young’s modulus (GPa)	13
	Capacitance (nF)	10.2
	Amount	16
**Workload**	Load 1 (g)	120
**Leaf spring**	Load 2 (g)	170
	Load 3 (g)	220
	Height (mm)	34
	Width (mm)	61
	Thickness (mm)	0.4
	Intersection angle	150°
	Material	Spring steel

**Table 2:** The peak power of all 16 piezoelectric layers.

Piezoelectric Layer Number	Current Voltage (V)	Power (μW)
**1**	19	591.8
**2**	2	6.6
**3**	5.2	44.3
**4**	4.8	37.8
**5**	8.2	110.2
**6**	2.2	7.9
**7**	4	26.2
**8**	7	80.3
**9**	17.2	485
**10**	2	6.6
**11**	7.2	85
**12**	6.4	67.1
**13**	6.2	63
**14**	2	6.6
**15**	4	26.2
**16**	7.4	89.8
